# Extraordinary Surgery

**Published:** 1893-05

**Authors:** 


EXTRAORDINARY SURGERY—A PATIENT SUCCESSFULLY OPERATED UPON AFTER BEING APPARENTLY DEAD.—The medical profession is much stirred, so far as it is as yet familiar with the facts in the case, by the details of a remarkable surgical operation performed within a few days by Dr. Albert Shunk, of New York. The fact accomplished is no less than the bringing back to life a person practically dead, as the term is usually understood, Among the many wonderful cases recorded in recent years probably not one has been marked by features as unique as that of which James McCaughrey is to-day the living example.

The patient, twelve years old, is the son of a New York merchant, lately deceased, living at No. 440 West Nineteenth street. The physician was called to the case late in the evening, and when he reached the house at 11 P. M., the patient had very marked symptoms of "appendicitis," or, as it is popularly called, inflammation of the blind intestine.

The diagnosis made plain the fact that the sufferer was doomed as far as could be determined by precedents in similar cases. On the advice of the physician the members of the family were advised of the gravity of the case, and when next morning a lump was observable over the seat of the inflammation the diagnosis was fully justified and the trouble was exactly stated to be an abscess around the vermiform appendix. An operation was at once recommended, but was delayed until the arrival of relatives who had been summoned in anticipation of death resulting.

It was 3 o'clock the next afternoon when the surgeon was finally called and directed to proceed. When he arrived it was discovered that the abscess had broken, thus covering the intestines with the liberated matter. In this crisis it was clear to the scientific mind that the boy was dying. The doctor told the family that dissolution would take place within an hour, and that though it was possible that an operation might result successfully, the chances were many times against it. Permission to proceed was given, it being understood that in event of failure to secure relief the end would not be hastened more than fifteen minutes.

The patient was quickly placed on a table, and while under the influence of a slight anæsthetic, an opening about eight inches long was made in the walls of the abdomen. From the incision there was at once a plenteous flow of pus. No sooner had this taken place than the pulse ceased to beat, the heart stopped, the jaw dropped and every evidence of the most complete collapse betokened the presence of death. The glassy eyes upturned, the coldness of the extremities and the death perspiration with which the body was covered left no doubt in the minds of those present that the experiment had resulted fatally.

At this point the surgeon's resourceful skill was displayed. With one hand he tore open the wound, while with the other he poured hot water from a pitcher into the abdominal cavity. For a brief interval the fluttering renewal of the action of the heart was apparent only to the practical medical eye, but soon the pulsations grew stronger and ere long the patient gave unmistakable signs of returning consciousness. Then the eyes were opened in a dazed sort of a way, as if the subject was awakening from a deep sleep. The action of the hot water having neutralized the effect of the ether that had been given, the patient was assisted to retain the ground gained by the administration of hyperdermic injections. With a new lease of life, there was a chance for the surgeon to look for the seat of the abscess. After several fruitless efforts to do this, it was decided to remove a portion of the intestines. For their better protection they were wrapped in a towel, warmed by dipping it into hot water, and placed on the table by the patient's side. Thus, with room to work, the walls of the cavity were explored, the abscess sack being found and successfully removed. Then the seat of the trouble was reached and the foreign substance which had caused the inflammation of the appendix was removed. The abdominal cavity was next thoroughly cleansed antiseptically, and after the pus had been washed from the exposed portions of the intestines they were returned to their place. Before this was done, however, a drainage tube of about eight inches in length was inserted so as to lead to the lower bowels. The wound was then closed and the sufferer left in charge of the surgeon's assistant and a trained nurse.

Remarkable as the operation is, it is still more marvelous that every day since the patient has gained in strength and is to-day convalescent. Frequent examinations have been made of the progress of affairs in the patient's abdomen, and as fast as expedient the drainage tube is withdrawn, until now only about three inches remain in service.

Operations for the removal of the "vermiform appendix" are by no means uncommon, but the surgeon's knife is rarely sanctioned by experienced practitioners in cases with physical conditions as extreme as those in the one here cited. Some idea of the interest manifested in the case may be gained from the statement that for several days efforts have been made to obtain the details without success. From points here given it is claimed that medical men now believe it to have been determined that no case of appendicitis need be necessarily regarded as fatal.

